# INFORMAL CARE NETWORK CHARACTERISTICS AND PSYCHOLOGICAL WELL-BEING AMONG CHINESE OLDER ADULTS

**DOI:** 10.1093/geroni/igae098.1604

**Published:** 2024-12-31

**Authors:** Jin You, Xinyue Shen, Yue Dong, Ziyi He

**Affiliations:** Wuhan University, Wuhan, Hubei, China (People’s Republic); Wuhan University, Wuhan, Hubei, China (People’s Republic); Queensland University of Techonology, Brisbane, Queensland, Australia; University of Washington Seattle, Seattle, Washington, United States

## Abstract

With population ageing, China is susceptible to heavy burdens related to caring for older people. With rural-to-urban migration, the availability of traditional family care has substantially declined. This study investigates the characteristics of informal care networks and their associations with mental health outcomes (i.e., depressive symptoms, life satisfaction) from social network perspective among Chinese middle-aged and older adults with care needs. Results from five waves (2011, 2013, 2015, 2018, and 2020) of the China Health and Retirement Longitudinal Study showed that, among 11,949 informal care receipts aged over 50 years, 62.83% received care from a spouse, 49.55% from children or grandchildren (with the mean size of 2.65), 3.78% from relatives (with the mean size of 3.61), and 5.13% from nonrelatives. The size of family care network (number of spouse, children or grandchildren, relatives), especially the number of children or grandchildren, predicted fewer depressive symptoms and greater life satisfaction. By contrast, care network diversity (number of care sources) predicted lower life satisfaction. Our findings suggest that Chinese older adults largely rely on and might psychologically benefit from a multiple-caregiver family network rather than on a single primary caregiver to address care needs. Further efforts should be made to understanding task sharing function, effective organization, and dynamic development of informal care network.

